# Influence of Ginsenoside Rh2 on Cardiomyocyte Pyroptosis in Rats with Acute Myocardial Infarction

**DOI:** 10.1155/2022/5194523

**Published:** 2022-10-12

**Authors:** Wanmei Song, Bin Dai, Yuntang Dai

**Affiliations:** ^1^Department of Cardiovascular Medicine, Longhui Country People's Hospital, Shaoyang 422200, Hunan, China; ^2^Clinical Laboratory, Longhui Country People's Hospital, Shaoyang 422200, Hunan, China

## Abstract

**Objective:**

This paper intends to verify through *in* vivo experiments whether ginsenoside Rh2 (G-Rh2) can play an anti-inflammatory role by modulating cardiomyocyte (CM) pyroptosis in rats with acute myocardial infarction (AMI), thereby alleviating myocardial injury.

**Methods:**

Twenty SD rats were randomized into control, L-Rh2, M-Rh2, and H-Rh2 groups, among which the latter three groups were modeled for AMI and given an intraperitoneal injection of G-Rh2 (L-Rh2: 2 mg/kg; M-Rh2: 4 mg/kg; H-Rh2: 8 mg/kg), while the control group was only treated with thoracotomy and sodium chloride injection. Heart rate (HR), systolic blood pressure (SBP), diastolic blood pressure (DBP), mean arterial pressure (MAP), left ventricular systolic pressure (LVSP), and left ventricular end-diastolic pressure (LVEDP) were recorded by ultrasonic diagnosis. Rats were killed under anesthesia, and the morphological characteristics of ventricular tissue were observed by electron microscope. Additionally, cardiac blood and ventricular tissues were collected to quantify the contents of myocardial injury markers (creatine phosphate kinase (CK), creatine phosphokinase-MB isoenzyme (CK-MB), and lactate dehydrogenase (LDH) by ELISA, as well as the expression of pyroptosis-related genes cysteinyl aspartate specific proteinase 1 (Caspase-1), gasdermin D (GSDMD), interleukin (IL)-1*β,* and NOD-like receptor thermal protein domain associated protein 3 (NLRP3) by qRT-PCR and Western blot).

**Results:**

Ultrasonic examination identified lower HR, SBP, DBP, MAP, and LVSP in the three Rh2 injection groups compared with the control group (*P* < 0.05); and in comparison with M- and H-Rh2 groups, HR, SBP, DBP, MAP, and LVSP were all lower in L-Rh2 group, while LVEDP was higher (*P* < 0.05). Microscopically, CMs and organelles in the L-RH2, M-RH2, and H-RH2 groups were damaged to varying degrees compared with the control group, with those in the L-RH2 group being the most serious. CK, CK-MB, and LDH were also the highest in the L-Rh2 group and the lowest in the control group, while their levels were obviously reduced in M- and H-Rh2 groups (*P* < 0.05). Finally, GSDMD, IL-1*β*, NLRP3, and Caspase-1 were found to be reduced in the control group, while pyroptosis-related gene expression in the M-Rh2 group was improved markedly (*P* < 0.05).

**Conclusion:**

G-Rh2 can inhibit the pathological development of AMI by relieving the focal death of CM and inhibiting the release of proinflammatory factors in the body, and the effect is significantly related to the dosage, which is expected to become a new treatment option for AMI in the future.

## 1. Introduction

Cardiovascular disease (CVD), with a predilection for middle-aged and elderly people, is a high-risk condition with mortality second only to malignancies [1]. Among them, acute myocardial infarction (AMI) is one of the most prevalent cardiovascular disorders, accounting for about 14–20% of the total CVD cases [2]. AMI is a disease primarily caused by acute coronary artery obstruction and myocardial necrosis, characterized by a high incidence, rapid progression, and high mortality [3]. According to statistics, there were over 2 million new AMI patients worldwide in 2019, an increase of about 7 times compared with a decade ago [4]. Moreover, the incidence of AMI also shows a trend of getting younger, with patients under 45 years old becoming increasingly common in recent years [5]. Meanwhile, as one of the high-risk types of CVD, the mortality rate of AMI has exceeded 60%, seriously threatening patients' life safety [6]. At present, the most effective clinical treatment for AMI is still coronary artery intervention or coronary artery bypass surgery. These two surgical methods are difficult to operate and invasive to some extent, and their application is limited for some patients. Moreover, due to the limitation of surgery, when a patient suffers from a sudden illness, emergency rescue cannot be performed by surgery, which undoubtedly increases the risk of adverse events [7]. Therefore, the search for an effective and rapid treatment for AMI has become a hotspot in modern clinical research, but no significant results have been achieved yet.

Undoubtedly, traditional Chinese medicine (TCM) is the safest therapy among all kinds of treatments in the field of modern medicine. However, as TCM treatment is generally a long-term and continuous therapy, it can hardly take effect quickly and is therefore rarely used in emergency treatment [8]. But by extracting the main active ingredients of TCM separately, their efficacy can be rapidly brought into play while ensuring the safety profile. Ginsenoside Rh2 (G-Rh2) is the hydrolyzed product of ginsenoside, the active component of ginseng. Compared with ginsenoside, Rh2 has higher purity and a faster drug metabolism rate [9]. At present, it has been found clinically that Rh2 can participate in the treatment of tumor diseases in multiple ways such as inhibiting tumor growth and reversing tumor cell drug resistance, with health and rehabilitation-promoting effects like improving human immune resistance and relieving fatigue [10, 11]. In a variety of CVD, Rh2 has also been found to have a good effect on reducing cardiac fibrosis, but its application value in AMI is still unclear [12]. We believe that Rh2 may also be expected to become a new treatment direction for AMI in the future, providing a more reliable guarantee for the life safety of AMI patients. Besides, Cellular apoptosis is a new programmed cell death, which is characterized by its dependence on inflammatory Caspase-1 and the release of a large number of proinflammatory factors through NLRP3 and other pathways. Cellular apoptosis is widely involved in the development of infectious diseases, nervous system-related diseases, and atherosclerotic diseases. Arterial injury and inflammation always run through the pathophysiological process of AMI progression to ventricular remodeling and heart failure. Therefore, cellular apoptosis is closely related to the development of AMI.

Consequently, this study intends to analyze the influence of Rh2 on cardiomyocytes (CMs) of AMI rats, aiming at confirming the effect and mechanism of Rh2 on AMI and laying a foundation and reference for future clinical application of Rh2.

## 2. Materials and Methods

### 2.1. Main Reagents

Western blot kit and BCA kit (Beijing Dingguo Changsheng Biotechnology); NLRP3, Caspase-1, GSDMD, IL-1*β*, and *β*-actin primary antibodies and HRP goat anti-rabbit IgG (ABclonal, USA); high-efficiencyenhanced-enhanced chemiluminescence (ECL) Kit (Genview, USA); TRIzol extraction kit (Thermo Fisher Scientific, USA); miRNA 1^st^ Strand cDNA Synthesis Kit and miRNA Universal SYBR qPCR Master Mix (Nanjing Vazyme Biotech); DEPC water (Shanghai Beyotime Biotechnology); ELISA kit (Wuhan Fine Biotech).

### 2.2. Rat Data

Twenty SPF SD rats ordered from Hunan SJA Laboratory Animal Co., Ltd. (Animal License Number: SYXK (Xiang) 2019–0017) were caged with 5 rats each and raised in an environment of 18–26°C, 40–70% humidity, and 12 : 12 h light-dark regime. They were given standard chow and allowed free access to water. This experiment was carried out in strict accordance with the Declaration of Helsinki and has been approved by the Animal Ethics Committee of our hospital (KYSQ 2020–07).

### 2.3. Grouping and Modeling

Twenty SD rats were randomized into 4 groups, each with 5 rats. Three groups were established as AMI model rats by referring to the study of Wu et al. [13]. Anesthesia was performed, and after trachea incision and intubation, an animal ventilator and ECG detection equipment were externally connected. The rat heart was then exposed by a tip of 3 cm in length along the gap between the third and fourth ribs. After confirming the position of the left anterior descending coronary artery at the junction of the pulmonary artery conical and left atrial appendage, ligation was performed 2 mm from the root of the left atrial appendage. If the anterior wall of the left ventricle was pale locally, the wall motion was weakened, and the ST segment of leads I and II of ECG was obviously increased, a syringe was used to extract the gas in the thoracic cavity. The chest was closed after the thoracic pressure was restored. The other group was treated as the control group only with thoracotomy. Then, three groups of AMI rats were intraperitoneally injected with 2 mg/kg (L-Rh2 group), 4 mg/kg (M-Rh2 group), and 8 mg/kg(H–Rh2 group) of Rh2, respectively, and the control group rats were given 0.9% sodium chloride injection intraperitoneally. The four groups of rats were continuously administered for 2 weeks.

### 2.4. Rat Hemodynamics and Cardiac Function Detection

Fourteen days after modeling, the rats were weighed, and the heart rate (HR), systolic/diastolic blood pressure (SBP/DBP), mean arterial pressure (MAP), left ventricular systolic pressure (LVSP), and left ventricular end-diastolic pressure (LVEDP) were examined by ultrasound under anesthesia. Then 3–5 ml of 10% potassium chloride was injected, the thorax was opened to take out the whole heart, and the right ventricle was cut off. The rest was dried with filter paper and weighed to calculate the left ventricular mass index.

### 2.5. Detection of Myocardial Injury Markers

The rat heart blood was collected into the coagulation-promoting tube and left standing for half an hour, and serum was obtained after 10 min of centrifugation (1500 × g, 4°C) to quantify creatine phosphate kinase (CK), creatine phosphokinase-MB isoenzyme (CK-MB) and lactate dehydrogenase (LDH) contents by ELISA. The testing process was strictly in accordance with the manufacturer's instructions.

### 2.6. CM Injury Determination

The rat's left ventricle was divided into three portions, one of which was placed into glutaraldehyde fixative and rinsed with a phosphate buffer solution at 4°C for 0.4–2.0 h. After fixation in osmic acid fixative for 2 hours, it was dehydrated by 50% ethanol for 15 min, 70% ethanol for 15 min, 80% ethanol for 15 min, 90% ethanol for 15 min, and 100% ethanol for 20–30 min. Following sufficient dehydration, it was soaked with an embedding agent first, and then with a new embedding agent for 30 min before embedding. Finally, it was sliced into 40–50 nm ultrathin slices and placed in a 3 mm diameter copper net for electron microscopy observation.

### 2.7. Pyroptosis-Related Gene Expression Detection

The other two portions of the left ventricular tissues were taken, one of which was extracted with a TRIzol extraction kit for total RNA. After purity verification by ultraviolet spectrophotometer, the RNA was reverse transcribed into cDNA. The sequences of primers used were designed and synthesized by Sangon Biotech, Shanghai (Table 1). ABI7500 quantitative PCR instrument was used for real-time quantitative PCR. The reaction procedure was 95°C for 5 min, and 30 cycles of 95°C for 5 s and 60°C for 30°s. High resolution melting: 95°C, 60°s; 55°C 30°s; and 95°C 30°s. NOD-like receptor thermal protein domain associated protein 3 (NLRP3), cysteinyl aspartate specific proteinase 1 (Caspase-1), gasdermin D (GSDMD), and interleukin (IL)-1*β* levels were normalized with *β*-actin and computed via 2^−ΔΔCt^. The last portion of the left ventricular sample was extracted by RIPA lysis buffer to retrieve the total protein, which was then quantified by BCA and subjected to SDS-PAGE gel electrophoresis. Following denaturation at 95°C for 10 min, cooling, sample loading, and electrophoresis, the protein was treated with membrane transfer, sealing, incubation with primary and secondary antibodies, as well as development with a chromogenic agent. The protein bands were finally observed by the gel image analysis system to determine NLRP3, Caspase-1, GSDMD, and IL-1*β* levels relative to *β*-actin or GAPDH. For the sequences of primers see Table 1.

### 2.8. Statistics and Methods

SPSS22.0 performed statistical analysis in this research. Each test in this study was run in duplicate, and the results were expressed as $\left(\bar{\chi }\pm s\right)$. Variance analysis and the Bonferroni test were used for multigroup and intragroup comparisons, respectively, with the difference, deemed significant when *P* < 0.05.

## 3. Results

### 3.1. Modeling Results

No peritonitis or death occurred in rats in this study. After 2 weeks of modeling, control rats were observed to be more active in diet and water intake, with shiny hair and certain mobility. However, L-Rh2 group rats showed no obvious signs of activity, dull hair color, and no self-feeding ability. In contrast, M-Rh2 group rats were occasionally active, and some could still eat autonomously. And the activity ability of H-Rh2 group rats was slightly lower compared with control rats, with the ability to eat independently.

### 3.2. Comparison of Hemodynamics and Cardiac Function

**Figure 1:**
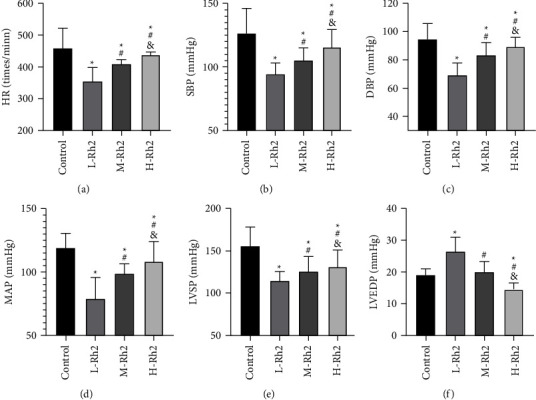
Comparison of hemodynamics and cardiac function. (a) Comparison of HR. (b) Comparison of SBP. (c) Comparison of DBP. (d) Comparison of MAP. (e) Comparison of LVSP. (f) Comparison of LVEDP. ^*∗*^: Compared with the control group, the difference was statistically significant (*P* < 0.05). ^#^: Compared with the L-Rh2 group, the difference was statistically significant (*P* < 0.05). &: Compared with the M-Rh2 group, the difference was statistically significant (*P* < 0.05).

Control rats presented higher HR, SBP, DBP, MAP, and LVSP levels than the three groups of rats injected with Rh2, as indicated by the Ultrasonic examination. And the control group had a LVEDP level similar to the M-Rh2 group (*P* > 0.05), which was lower compared with the L-Rh2 group and higher than the H-Rh2 group (*P* < 0.05). A multigroup comparison of the three groups of rats injected with Rh2 revealed lower HR, SBP, DBP, MAP, and LVSP and higher LVEDP in the L-Rh2 group compared with M- and H-Rh2 groups (*P* < 0.05). Likewise, the M-Rh2 group had lower HR, SBP, DBP, MAP, and LVSP while higher LVEDP than the H–Rh2 group (Figure 1).

### 3.3. Comparison of Myocardial Injury Markers

**Figure 2:**
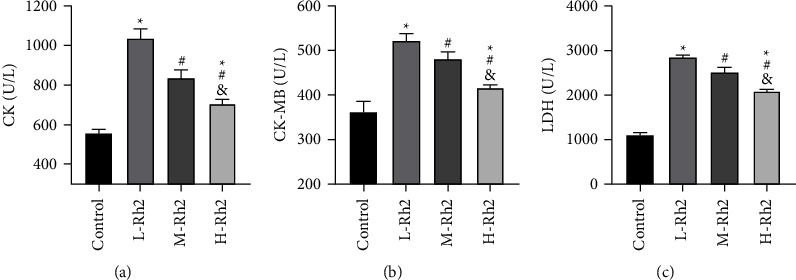
Comparison of myocardial injury markers. (a) Comparison of CK. (b) Comparison of CK-MB. (c) Comparison of LDH. ^*∗*^: Compared with the control group, the difference was statistically significant (*P* < 0.05). ^#^: Compared with the L-Rh2 group, the difference was statistically significant (*P* < 0.05). &: Compared with the M-Rh2 group, the difference was statistically significant (*P* < 0.05).

Among the four groups, serum CK, CK-MB, and LDH contents were the lowest in the control group and the highest in the L-Rh2 group (*P* < 0.05). And in comparison with H–Rh2 group, CK, CK-MB, and LDH were higher in the M-Rh2 group (*P* < 0.05) (Figure 2).

### 3.4. Comparison of CM Injury

**Figure 3:**
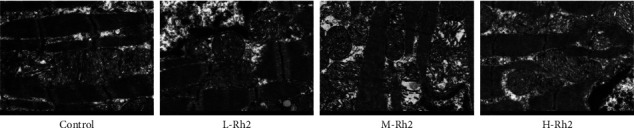
Electron microscopy was used to observe myocardial injury.

As indicated by the electron microscopic examination, the ultrastructure of CMs in the control group was basically intact, with a clear structure and no obvious apoptotic bodies observed. L-Rh2 group rats exhibited obviously damaged and seriously broken CMs, with visible apoptotic bodies as well as disordered and partially broken myofibrils, and the degree of injury in the L-Rh2 group was significantly higher than that in the M-Rh2 group and H–Rh2 group. M-Rh2 group showed relatively intact CM morphology, with a small number of nuclei destroyed, slight swelling of mitochondrial aggregation, partial disappearance of mitochondrial ridges, local vacuoles, and occasional apoptotic bodies. In the H-RH2 group, the morphological structure of CM was the most intact of the three modeling groups, and its structural characteristics were similar to that of the control group (Figure 3).

### 3.5. CM Pyroptosis-Related Gene Expression

**Figure 4:**
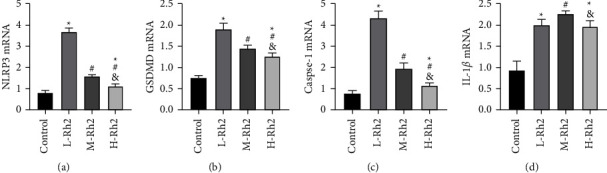
CM pyroptosis-related gene expression. (a) Comparison of NLRP3 mRNA. (b) Comparison of GSDMD mRNA. (c) Comparison of Caspase-1 mRNA. (d) Comparison of IL-1*β* mRNA. ^*∗*^: Compared with the control group, the difference was statistically significant (*P* < 0.05). ^#^: Compared with the L-Rh2 group, the difference was statistically significant (*P* < 0.05). &: Compared with the M-Rh2 group, the difference was statistically significant (*P* < 0.05).

qPCR results determined lower GSDMD, IL-1*β*, NLRP3, and Caspase-1 mRNA levels in the control group compared with the three Rh2 intervention groups (*P* < 0.05). The multigroup comparison of GSDMD, NLRP3, and Caspase-1 mRNA identified that the L-Rh2 group had the highest levels while the H-Rh2 group had the lowest levels (*P* < 0.05). However, no statistical significance was found in IL-1*β* mRNA between L- and H–Rh2 groups (*P* > 0.05), while the IL-1*β* mRNA in L- and H–Rh2 groups were lower than that of the M-Rh2 group (*P* < 0.05) (Figure 4).

### 3.6. CM Pyroptosis-Related Protein Expression

**Figure 5:**
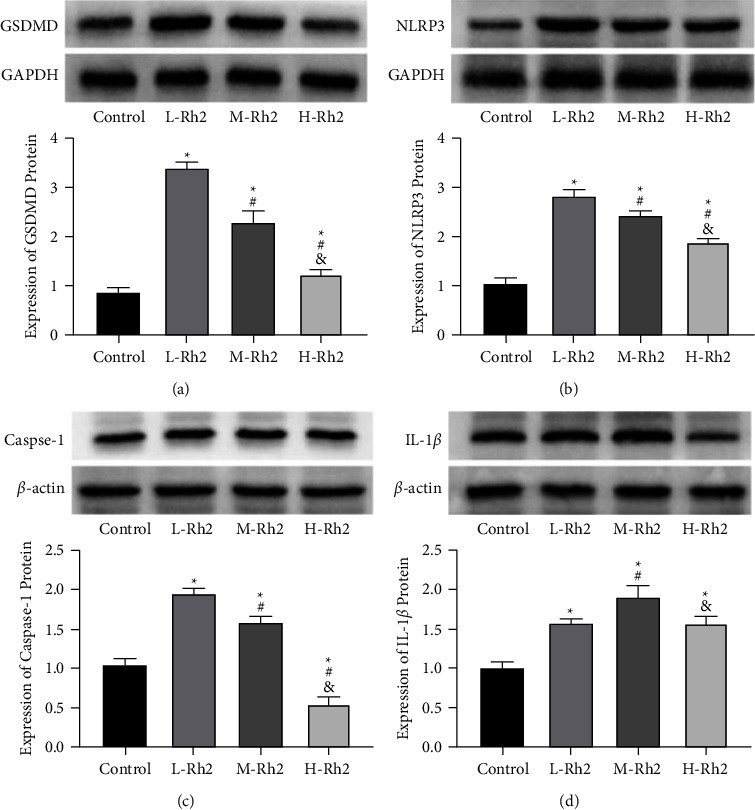
CM pyroptosis-related protein expression. (a) Comparison of GSDMD protein expression. (b) Comparison of NLRP3 protein expression. (c) Comparison of Caspase-1 protein expression. (d) Comparison of IL-1*β* protein expression. ^*∗*^: Compared with the control group, the difference was statistically significant (*P* < 0.05). ^#^: Compared with the L-Rh2 group, the difference was statistically significant (*P* < 0.05). &: Compared with the M-Rh2 group, the difference was statistically significant (*P* < 0.05).

At last, the Western blot analysis showed lower GSDMD and IL-1*β* protein while higher NLRP3 and Caspase-1 protein levels in the control group compared with L-, M-, and H-Rh2 groups (*P* < 0.05). Among the three groups treated with Rh2 injection, L-Rh2 had the lowest GSDMD, NLRP3, and Caspase-1 levels, while the H-Rh2 group showed lower levels than the M-Rh2 group (*P* < 0.05) (Figure 5).

## 4. Discussion

At present, the high incidence and mortality of CVD have caused a great burden on global medical care, among which AMI is one of the primary causes of death [14]. Therefore, improving the treatment level of AMI and controlling its incidence have become a research focus in the cardiovascular field. As a new type of programmed cell death, apoptosis has been confirmed to participate in the pathogenesis and progression of AMI by regulating the inflammatory response and stimulating the release of proinflammatory factors, but the exact mechanism has not yet been clearly elucidated [15]. Rh2, the main active component of ginseng, has shown excellent effects in multiple pathological improvements, such as antitumor, antiallergy, anti-inflammation, enhancing immunity, and resisting hypoxia, in addition to a certain protective role in CVD [16–19]. Therefore, this paper intends to verify in vivo whether Rh2 can exert anti-inflammatory action by regulating CM pyroptosis in AMI rats, thus alleviating myocardial injury and providing a reliable theoretical basis for future treatment of AMI.

Compared with the control group, a number of indicators of cardiac function and hemodynamics were found to be abnormal in rats after AMI modeling, while these indicators of AMI rats recovered after Rh2 administration, among which the recovery effect of the H-RH2 group was the most significant, indicating that Rh2 has the effect of alleviating AMI. In previous studies, we found that Rh2 can validly reduce nerve damage caused by cerebral ischemia and cardiotoxicity after breast cancer [20, 21], which can also preliminarily verify our results and confirm the improvement effect of Rh2 on CVD. Moreover, under electron microscope observation of left ventricular tissue, we found seriously damaged rat CMs and organelles post-AMI modeling, which were later significantly improved with the increase of Rh2 dosage. Similarly, the decrease of CK, CK-MB, and LDH contents in the H-Rh2 group can also verify our view. Past evidence has shown that Rh2 has a stable regulatory effect on cell pyroptosis in diabetic nephropathy [22]. Therefore, we detected pyroptosis-related genes in four groups of rats. The results were also in line with our expectations, that is, the control group had lower GSDMD, IL-1*β*, NLRP3, and Caspase-1 levels, while pyroptosis-related gene expression was improved in the three groups of rats injected with RH2, with the most significant improvement found in H-Rh2 group. This demonstrates that Rh2 can relieve CM scorch and inhibit the progress of AMI, and its effect is related to the dosage used. As the main executor of pyroptosis, GSDMD is mainly hydrolyzed by Caspase to release a large quantity of inflammatory factors, promoting cell membrane rupture [23]. NLRP3, being a member of the pattern recognition receptor family, is directly induced by many external stimuli (such as ischemia), which in turn activates Pro-Caspase-1 into Caspase-1 to bind to IL-1*β* precursor, resulting in the increase of synthesis and secretion of IL-1*β* and TNF-*α* and thus participating in immunoreaction [24]. Integrating previous literature and the findings we obtained, we believe that NLRP3 is activated when CMs injury occurs in rats. while activating Caspase to promote CM scorch death and inflammation release, NLRP3 can also directly induce the synthesis of inflammatory factors and inhibit the immune function of patients, and finally lead to the occurrence of AMI through a variety of pathways. And Rh2 can directly block the activation of NLRP3, inhibit the aggravation and expansion of the body's inflammatory response, and repair the above pathological changes, thereby achieving the purpose of relieving and treating AMI.

In previous studies, Rh2 has also been found to modulate the oxidative stress response of nerve tissue and the autophagy capacity of cells [25, 26], which may also be the mechanism by which Rh2 influences AMI. Hence, it is worthwhile to carry out more in vitro experiments to confirm. In addition, due to the differences between experimental animals and humans, the specific clinical effect of Rh2 in AMI needs to be confirmed through clinical trials. In addition, we compared different doses in this study, and the highest dose appeared to be the best. Therefore, whether further increases in doses can achieve a better effect and the optimal dose threshold of Rh2 need to be further studied. In order to obtain the most convincing experimental results, we will conduct a more comprehensive experimental analysis as soon as possible to solve the above limitation.

To sum up, G-Rh2 can inhibit the pathological development of AMI by relieving CM pyroptosis and is expected to become a new treatment option for AMI in the future, providing a more reliable safety guarantee for patients.

## Figures and Tables

**Table 1:** Primer sequences.

Gene name	F (5′–3′)	R (5′–3′)
GSDMD	TTGAGTGTCTGGTGCTCGAC	ATGGGGTGCTCTGTTCCAAG
NLRP3	GGTGACCTTGTGTGTGCTTG	ATGTCCTGAGCCATGGAAGC
IL-1*β*	GGTTCAAGGCATAACAGGCTC	TCTGGACAGCCCAAGTCAAG
Caspase-1	TTATCAGGGTTGACCCCTTGG	TTGCCCTCAGGATCTTGTCAG
GAPDH	ACACGAGTCCTGGTGACTTTG	GGGCTTAGGTCCACACAGAA

## Data Availability

The data used and/or analyzed during the current study are available from the corresponding author.
